# Bis[(*E*)-4-chloro-2-(2-furylmethyl­imino­meth­yl)phenolato]iron(II)

**DOI:** 10.1107/S1600536808015559

**Published:** 2008-05-30

**Authors:** Dong-Sheng Xia, Wu Chen, Jing Huang, Qing-Fu Zeng

**Affiliations:** a Engineering Research Center for Clean Production of Textile Printing, Ministry of Education, Wuhan University of Science and Engineering, Wuhan 430073, People’s Republic of China

## Abstract

The Fe atom of the title compound, [Fe(C_12_H_9_ClNO_2_)_2_], lies on a crystallographic twofold rotation axis. The Fe^II^ atom is four-coordinated in a tetra­hedral geometry by the O and N atoms of the two Schiff base ligands. The O atom of the furan substituent in the ligand unit is not involved in coordination to the Fe atom.

## Related literature

For related structures, see: Chen & Wang (2006 ▶); Chen *et al.* (2007 ▶); Ran *et al.* (2006 ▶); Ye *et al.* (2007 ▶); Zhu *et al.* (2003 ▶).
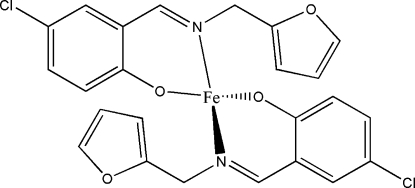


## Experimental

### 

#### Crystal data

[Fe(C_12_H_9_ClNO_2_)_2_]
*M* *_r_* = 525.15
Monoclinic, 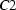

*a* = 22.550 (4) Å
*b* = 4.6270 (6) Å
*c* = 13.822 (3) Å
β = 127.73 (3)°
*V* = 1140.6 (4) Å^3^
*Z* = 2
Mo *K*α radiation
μ = 0.93 mm^−1^
*T* = 298 (2) K
0.21 × 0.21 × 0.20 mm

#### Data collection

Bruker SMART CCD area-detector diffractometer
Absorption correction: multi-scan (*SADABS*; Sheldrick, 1996 ▶) *T*_min_ = 0.829, *T*_max_ = 0.836
1314 measured reflections
1262 independent reflections
973 reflections with *I* > 2σ(*I*)
*R*_int_ = 0.035

#### Refinement

*R*[*F* ^2^ > 2σ(*F* ^2^)] = 0.060
*wR*(*F* ^2^) = 0.203
*S* = 1.06
1262 reflections
151 parameters
1 restraint
H-atom parameters constrained
Δρ_max_ = 0.60 e Å^−3^
Δρ_min_ = −0.40 e Å^−3^
Absolute structure: Flack (1983 ▶), with no Friedel pairs
Flack parameter: −0.02 (9)

### 

Data collection: *SMART* (Bruker, 1998 ▶); cell refinement: *SAINT* (Bruker, 1998 ▶); data reduction: *SAINT*; program(s) used to solve structure: *SHELXS97* (Sheldrick, 2008 ▶); program(s) used to refine structure: *SHELXL97* (Sheldrick, 2008 ▶); molecular graphics: *SHELXTL* (Sheldrick, 2008 ▶); software used to prepare material for publication: *SHELXTL* .

## Supplementary Material

Crystal structure: contains datablocks global, I. DOI: 10.1107/S1600536808015559/sj2505sup1.cif

Structure factors: contains datablocks I. DOI: 10.1107/S1600536808015559/sj2505Isup2.hkl

Additional supplementary materials:  crystallographic information; 3D view; checkCIF report

## Figures and Tables

**Table:** 

Fe1—O1	1.888 (8)
Fe1—N1	1.992 (8)

**Table:** 

O1—Fe1—O1^i^	124.0 (6)
O1—Fe1—N1	95.2 (3)
O1—Fe1—N1^i^	113.5 (3)
N1—Fe1—N1^i^	117.3 (5)

Symmetry code: (i) 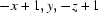.

**Fig. 1.**
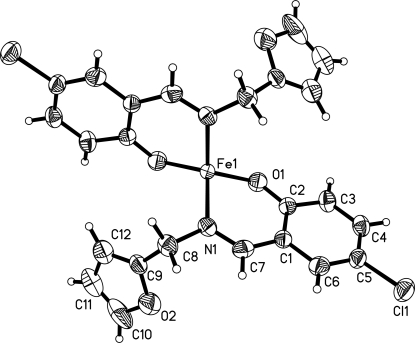
The structure of (I) showing 30% probability displacement ellipsoids and the atom-numbering scheme. Numbered atoms are related to un-numbered atoms by the symmetry code 1-x, y, 1-z.

## supplementary crystallographic information

### Comment

As part of our ongoing interest in the structure of iron complexes (Zhu *et al.*, 2003), we report herein the crystal structure of the title compound, a new mononuclear iron(II) complex, (I), Fig. 1, derived from the Schiff base ligand 4-chloro-2-[(furan-2-ylmethylimino)methyl]phenol.

Compound (I) possesses crystallographic two-fold symmetry. The Fe^II^ atom in (I) is four-coordinate in a tetrahedral geometry, binding to the O and N atoms of two Schiff base ligands. The O atom of the furan substituent in the ligand lies well away from the coordination sphere of the Fe atom. The coordinate bond values (Table 1) are comparable to values observed in other iron(II) complexes (Chen & Wang, 2006; Chen *et al.*, 2007; Ran *et al.*, 2006; Ye *et al.*, 2007).

### Experimental

5-Chlorosalicylaldehyde (62.4 mg, 0.2 mmol), furan-2-ylmethylamine (19.4 mg, 0.2 mmol), and FeCl_2_ (12.6 mg, 0.1 mmol) were dissolved in methanol (30 ml). The mixture was stirred for 30 min at room temperature. The resulting solution was kept still in air for a few days, yielding brown crystals.

### Refinement

H atoms were placed in idealized positions and constrained to ride on their parent atoms with C–H distances in the range 0.93–0.97 Å, and with *U*_iso_(H) set at 1.2*U*_eq_(C).

### Crystal data

**Table:**

[Fe(C_12_H_9_ClNO_2_)_2_]	*F*_000_ = 536
*M**_r_* = 525.15	*D*_x_ = 1.529 Mg m^−^^3^
Monoclinic, *C*2	Mo *K*α radiation λ = 0.71073 Å
Hall symbol: C 2y	Cell parameters from 823 reflections
*a* = 22.550 (4) Å	θ = 2.4–26.2º
*b* = 4.6270 (6) Å	µ = 0.93 mm^−^^1^
*c* = 13.822 (3) Å	*T* = 298 (2) K
β = 127.73 (3)º	Block, brown
*V* = 1140.6 (4) Å^3^	0.21 × 0.21 × 0.20 mm
*Z* = 2	

### Data collection

**Table:**

Bruker SMART CCD area-detector diffractometer	1262 independent reflections
Radiation source: fine-focus sealed tube	973 reflections with *I* > 2σ(*I*)
Monochromator: graphite	*R*_int_ = 0.035
*T* = 298(2) K	θ_max_ = 26.0º
ω scans	θ_min_ = 1.9º
Absorption correction: multi-scan(SADABS; Sheldrick, 1996)	*h* = −21→27
*T*_min_ = 0.829, *T*_max_ = 0.836	*k* = −5→0
1314 measured reflections	*l* = −17→0

### Refinement

**Table:**

Refinement on *F*^2^	Hydrogen site location: inferred from neighbouring sites
Least-squares matrix: full	H-atom parameters constrained
*R*[*F*^2^ > 2σ(*F*^2^)] = 0.060	*w* = 1/[σ^2^(*F*_o_^2^) + (0.1149*P*)^2^ + 2.2051*P*] where *P* = (*F*_o_^2^ + 2*F*_c_^2^)/3
*wR*(*F*^2^) = 0.203	(Δ/σ)_max_ = 0.001
*S* = 1.06	Δρ_max_ = 0.60 e Å^−^^3^
1262 reflections	Δρ_min_ = −0.40 e Å^−^^3^
151 parameters	Extinction correction: SHELXL97 (Sheldrick, 2008), Fc^*^=kFc[1+0.001xFc^2^λ^3^/sin(2θ)]^-1/4^
1 restraint	Extinction coefficient: 0.011 (3)
Primary atom site location: structure-invariant direct methods	Absolute structure: Flack (1983), 0 Friedel pairs
Secondary atom site location: difference Fourier map	Flack parameter: −0.02 (9)

### Special details

**Table:**

Geometry. All esds (except the esd in the dihedral angle between two l.s. planes) are estimated using the full covariance matrix. The cell esds are taken into account individually in the estimation of esds in distances, angles and torsion angles; correlations between esds in cell parameters are only used when they are defined by crystal symmetry. An approximate (isotropic) treatment of cell esds is used for estimating esds involving l.s. planes.
Refinement. Refinement of F^2^ against ALL reflections. The weighted R-factor wR and goodness of fit S are based on F^2^, conventional R-factors R are based on F, with F set to zero for negative F^2^. The threshold expression of F^2^ > 2sigma(F^2^) is used only for calculating R-factors(gt) etc. and is not relevant to the choice of reflections for refinement. R-factors based on F^2^ are statistically about twice as large as those based on F, and R- factors based on ALL data will be even larger.

### Fractional atomic coordinates and isotropic or equivalent isotropic displacement parameters (Å^2^)

**Table:**

	*x*	*y*	*z*	*U*_iso_*/*U*_eq_	
Fe1	0.5000	0.5044 (3)	0.5000	0.0419 (6)	
Cl1	0.87863 (17)	0.7649 (11)	1.0179 (3)	0.0929 (13)	
N1	0.5246 (5)	0.728 (2)	0.6435 (8)	0.055 (2)	
O1	0.5933 (4)	0.313 (2)	0.5865 (7)	0.067 (2)	
O2	0.4619 (6)	0.600 (3)	0.7844 (9)	0.102 (4)	
C1	0.6584 (5)	0.630 (3)	0.7611 (9)	0.053 (2)	
C2	0.6554 (5)	0.419 (2)	0.6848 (9)	0.053 (3)	
C3	0.7253 (6)	0.309 (3)	0.7192 (11)	0.069 (3)	
H3	0.7250	0.1686	0.6709	0.083*	
C4	0.7929 (6)	0.406 (3)	0.8218 (11)	0.068 (3)	
H4	0.8377	0.3317	0.8429	0.081*	
C5	0.7933 (6)	0.620 (3)	0.8943 (10)	0.065 (3)	
C6	0.7284 (6)	0.737 (3)	0.8663 (10)	0.070 (3)	
H6	0.7300	0.8824	0.9145	0.084*	
C7	0.5937 (6)	0.759 (3)	0.7390 (10)	0.066 (3)	
H7	0.6021	0.8786	0.8003	0.079*	
C8	0.4677 (6)	0.890 (3)	0.6455 (11)	0.067 (3)	
H8A	0.4926	1.0336	0.7100	0.080*	
H8B	0.4329	0.9882	0.5681	0.080*	
C9	0.4259 (6)	0.683 (3)	0.6671 (10)	0.061 (3)	
C10	0.4146 (12)	0.427 (6)	0.786 (2)	0.137 (10)	
H10	0.4242	0.3498	0.8560	0.164*	
C11	0.3509 (10)	0.381 (4)	0.6710 (18)	0.105 (6)	
H11	0.3109	0.2606	0.6466	0.126*	
C12	0.3594 (7)	0.555 (4)	0.5982 (13)	0.087 (5)	
H12	0.3238	0.5781	0.5141	0.104*	

### Atomic displacement parameters (Å^2^)

**Table:**

	*U*^11^	*U*^22^	*U*^33^	*U*^12^	*U*^13^	*U*^23^
Fe1	0.0384 (9)	0.0462 (11)	0.0399 (9)	0.000	0.0233 (7)	0.000
Cl1	0.0661 (17)	0.122 (3)	0.0664 (18)	−0.012 (2)	0.0279 (15)	0.017 (2)
N1	0.062 (5)	0.047 (5)	0.065 (5)	0.000 (5)	0.044 (4)	−0.005 (4)
O1	0.065 (4)	0.068 (6)	0.066 (4)	−0.001 (4)	0.040 (4)	−0.017 (4)
O2	0.093 (6)	0.136 (12)	0.089 (6)	0.016 (7)	0.062 (5)	0.022 (7)
C1	0.052 (5)	0.054 (6)	0.053 (5)	0.001 (5)	0.032 (5)	0.006 (5)
C2	0.053 (5)	0.050 (8)	0.053 (5)	0.005 (5)	0.031 (5)	0.009 (5)
C3	0.061 (6)	0.062 (8)	0.088 (8)	−0.007 (6)	0.048 (6)	−0.008 (7)
C4	0.056 (6)	0.070 (9)	0.081 (7)	0.012 (6)	0.043 (6)	0.019 (7)
C5	0.048 (6)	0.081 (9)	0.055 (6)	0.001 (6)	0.026 (5)	0.013 (6)
C6	0.071 (7)	0.077 (9)	0.059 (6)	−0.001 (7)	0.038 (6)	0.010 (7)
C7	0.074 (7)	0.067 (8)	0.054 (6)	−0.004 (7)	0.038 (6)	0.002 (6)
C8	0.070 (7)	0.059 (7)	0.079 (7)	0.019 (6)	0.049 (6)	0.009 (6)
C9	0.061 (6)	0.067 (8)	0.070 (7)	0.011 (6)	0.048 (6)	0.005 (6)
C10	0.161 (17)	0.15 (3)	0.199 (19)	0.037 (18)	0.158 (17)	0.06 (2)
C11	0.112 (12)	0.085 (11)	0.167 (17)	0.000 (11)	0.110 (13)	−0.004 (13)
C12	0.080 (8)	0.088 (14)	0.102 (9)	−0.001 (9)	0.061 (8)	−0.007 (9)

### Geometric parameters (Å, °)

**Table:**

Fe1—O1	1.888 (8)	C3—H3	0.9300
Fe1—O1^i^	1.888 (8)	C4—C5	1.402 (17)
Fe1—N1	1.992 (8)	C4—H4	0.9300
Fe1—N1^i^	1.992 (8)	C5—C6	1.377 (16)
Cl1—C5	1.746 (12)	C6—H6	0.9300
N1—C7	1.294 (13)	C7—H7	0.9300
N1—C8	1.499 (12)	C8—C9	1.493 (16)
O1—C2	1.310 (12)	C8—H8A	0.9700
O2—C10	1.34 (2)	C8—H8B	0.9700
O2—C9	1.352 (14)	C9—C12	1.328 (18)
C1—C2	1.411 (14)	C10—C11	1.35 (2)
C1—C7	1.424 (15)	C10—H10	0.9300
C1—C6	1.429 (15)	C11—C12	1.39 (2)
C2—C3	1.431 (15)	C11—H11	0.9300
C3—C4	1.374 (16)	C12—H12	0.9300
			
O1—Fe1—O1^i^	124.0 (6)	C4—C5—Cl1	119.4 (9)
O1—Fe1—N1	95.2 (3)	C5—C6—C1	118.1 (13)
O1^i^—Fe1—N1	113.5 (3)	C5—C6—H6	121.0
O1—Fe1—N1^i^	113.5 (3)	C1—C6—H6	121.0
O1^i^—Fe1—N1^i^	95.2 (3)	N1—C7—C1	127.6 (11)
N1—Fe1—N1^i^	117.3 (5)	N1—C7—H7	116.2
C7—N1—C8	115.9 (9)	C1—C7—H7	116.2
C7—N1—Fe1	120.0 (8)	C9—C8—N1	109.7 (10)
C8—N1—Fe1	123.9 (7)	C9—C8—H8A	109.7
C2—O1—Fe1	123.4 (7)	N1—C8—H8A	109.7
C10—O2—C9	106.7 (14)	C9—C8—H8B	109.7
C2—C1—C7	123.7 (10)	N1—C8—H8B	109.7
C2—C1—C6	121.3 (10)	H8A—C8—H8B	108.2
C7—C1—C6	115.0 (11)	C12—C9—O2	108.5 (12)
O1—C2—C1	124.5 (9)	C12—C9—C8	135.8 (12)
O1—C2—C3	118.3 (10)	O2—C9—C8	115.7 (11)
C1—C2—C3	117.3 (9)	O2—C10—C11	111.3 (16)
C4—C3—C2	121.8 (12)	O2—C10—H10	124.4
C4—C3—H3	119.1	C11—C10—H10	124.4
C2—C3—H3	119.1	C10—C11—C12	103.7 (16)
C3—C4—C5	119.1 (10)	C10—C11—H11	128.2
C3—C4—H4	120.5	C12—C11—H11	128.2
C5—C4—H4	120.5	C9—C12—C11	109.7 (14)
C6—C5—C4	122.4 (11)	C9—C12—H12	125.2
C6—C5—Cl1	117.9 (11)	C11—C12—H12	125.2

Symmetry codes: (i) −*x*+1, *y*, −*z*+1.
